# Oral Hygiene

**Published:** 1906-08

**Authors:** J. F. Blalock

**Affiliations:** Ocala, Fla.


### ORAL HYGIENE.

J. F. Blalock, D. D. S., Ocala, Fla.

(Florida State Dental Society, 1906.)

This subject is one upon which much has been said, but it is one that is of so much importance that it should be kept before the mind of the dentist. Dentistry is no longer considered among the trades, but is recognized as a noble profession, and any man may well be proud to be classed among the ethical practitioners of this profession! Then, gentlemen, let me urge you to apply all your skill and knowledge toward making the mouths of your patients as nearly aseptic as lies within your power. Are we not sometimes a little careless upon this line? Crowns that do not fit—bridges that cannot be kept clean—we see them nearly every day.

The methods for rendering a mouth healthy are numerous, and the medicinal remedies without number. The secret lies in the diagnosis of the case—if your diagnosis is correct, your knowledge of materia medica and therapeutics will prompt the right mode of treatment. I believe the time will come when the progressive dentist will have a chemical laboratory in his office, where the saliva will be tested—cultures will be made from the oral bacteria, and oral hygiene will be treated scientifically. The health of the mouth depends upon:

1st. The skill of the dentist.

2d. The care of the patient.

3d. The skill of the physician.

Do you ask why I put the skill of the dentist first? Because we are living in a progressive age, and the average patient consults the dentist as early as possible—the average mother brings the little ones to you for your advice and care. And so many dentists do not give the deciduous teeth the care they should have—they extract them, instead of treating and filling them, thus causing irregularities in the permanent teeth. The average mother is only too willing to pay for the work, when once you explain why it should be done; and as soon as you win the confidence of the little one, by simple operations, first it is wonderful what good patients they become.

Then it is our duty to place the mouth in an aseptic condition by our operative skill and mechanism, aided by such antiseptics and germicides as our knowledge of therapeutics and bacteriology may suggest,—instructing the patient as to the future care of the teeth. In the second place, if you succeed in getting them interested in the teeth, and get them to promise to come to your office every sixty days, the victory is half won. I have patients who come at regular intervals for examinations, they become interested, and before leaving the office they put the date down in a memorandum book, so they will not forget the time to come again. In the last place —we all realize the hereditary and constitutional causes of septic conditions of the mouth, and if the patient needs constitutional treatment we should urge them to engage the services of their family physician,—as I do not believe in infringing too much upon our brother professional man.—the physician.

Then let us keep this subject upon our minds, and conscientiously construct our work so it will be of an aseptic nature, and instruct our patients to the best of our ability and strive to make our ability the best. Then, when our life's work is ended, may we all receive that welcome plaudit: "Well done, thou good and faithful servant."

